# Supplementary material for: The Rho/Rac Guanine Nucleotide Exchange Factor Vav1 Regulates Hif-1α and Glut-1 Expression and Glucose Uptake in the Brain
Source: Int J Mol Sci. 2020 Feb 17;21(4):1341. doi: 10.3390/ijms21041341 (PMC7072975; doi:10.3390/ijms21041341)
Supplement: Supplementary file 1 [file ijms-21-01341-s001.pdf]

Supplementary Material

Supplementary Figures

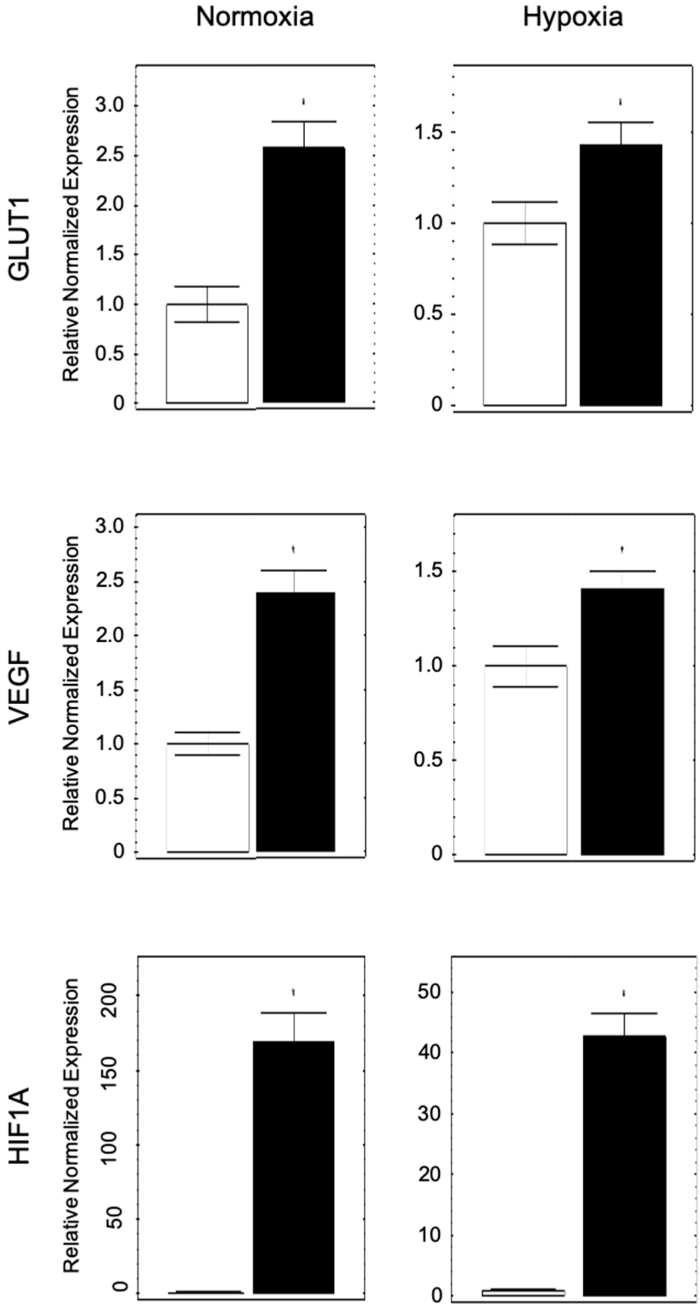

Supplementary Figure 1. HUVEC were transfected with empty vector (blank bar) or HIF-1α (filled bar). Cells were incubated in normoxia or hypoxia (1% O2) for 16 hours. GLUT-1, VEGF, and HIF-1α mRNA were measured by RT-qPCR. Mean ± SD, *p < 0.05.

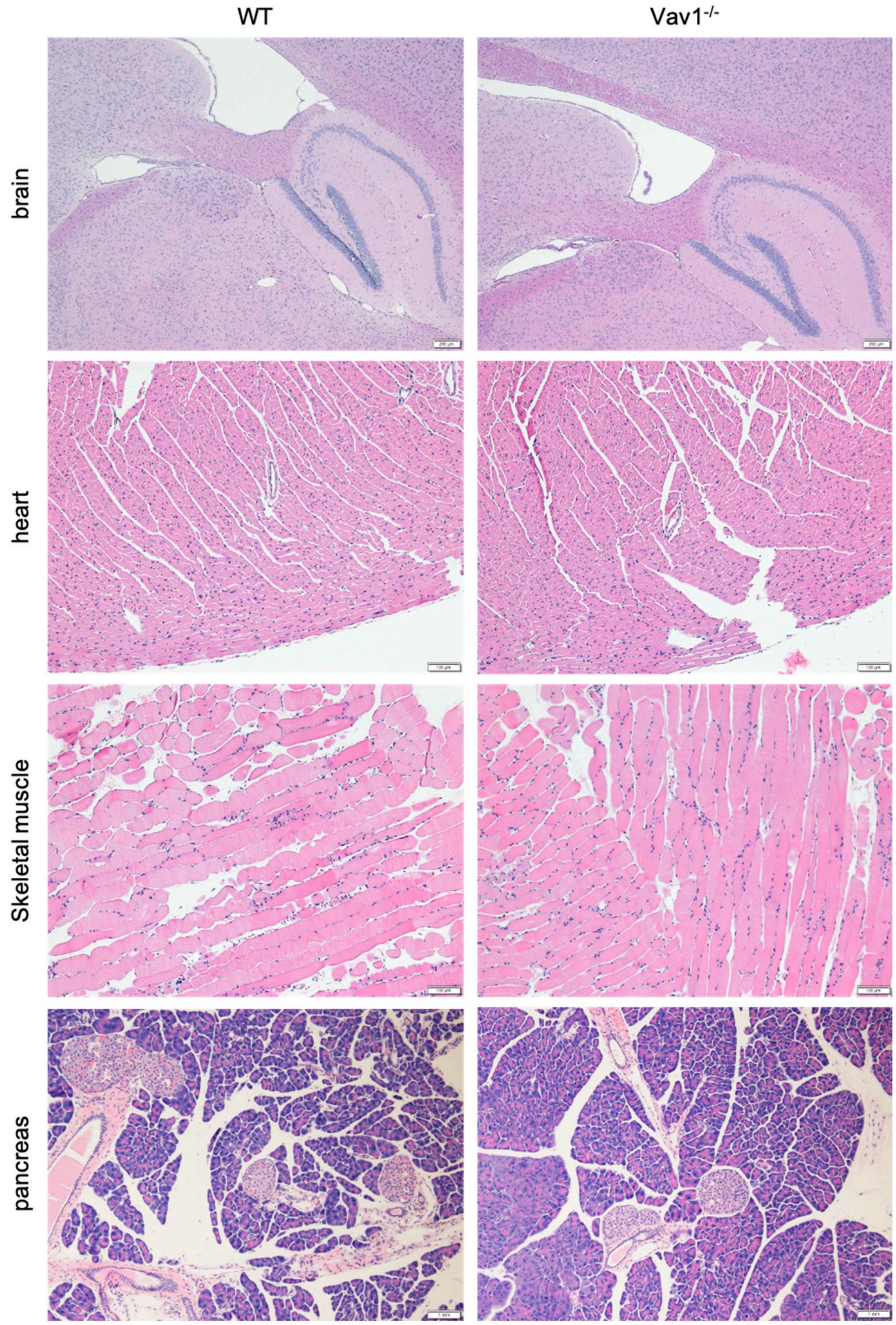

Supplementary Figure 2. Brain, heart, skeletal muscle, and pancreas were obtained from 5 wild type and 5 endothelial Vav1-deficient mice after perfusion. Tissues were fixed with 4% paraformaldehyde and then embedded in paraffin. Slides were stained with hematoxylin and eosin to visualize the basal structure of organs. Representative figures are shown. (Scale Bar, Brain 200 μm, heart, skeletal muscle, pancreas 100 μm)

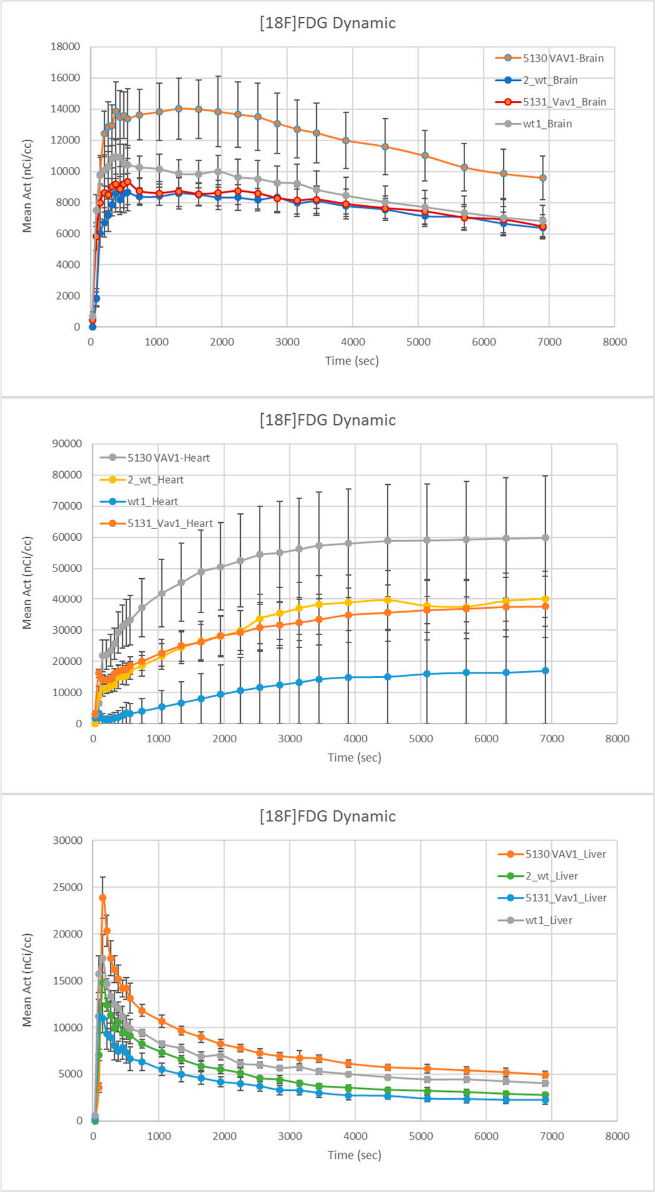

Supplementary Figure 3. The kinetic curves of ^{18}F-FDG after the intravenous injection. After the injection of 200 μCi, the level of ^{18}F-FDG was measured from the brain (top), heart (middle) and liver (bottom). Animals were fasted (standard clinical protocol) for 12 hours prior to injection. Animals were anesthetized for the hour uptake phase (static imaging).

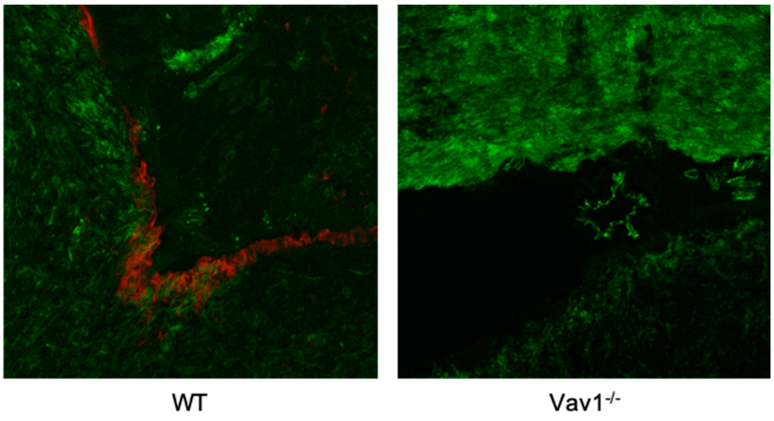

Supplementary Figure 4. Vav1 deficiency was validated from the endothelial specific Vav1 deficient mouse. Compared to wild type mouse, Vav1 (red) expression was abrogated in the endothelial specific Vav1 deficient mouse brain. Neurons were stained with neurofilament antibody (green). The image size is 425 μm X 425 μm.
